# From Bottom-Up Approaches to Levels of Organization and Extended Critical Transitions

**DOI:** 10.3389/fphys.2012.00232

**Published:** 2012-07-17

**Authors:** Giuseppe Longo, Maël Montévil, Arnaud Pocheville

**Affiliations:** ^1^Centre National de la Recherche Scientifique, Centre de Recherche en Épistémologie Appliquée – PolytechniqueParis, France; ^2^École Normale SupérieureCIRPHLESS, Paris, France; ^3^École doctorale 474 FDV, Paris 5 DescartesParis, France; ^4^Soto and Sonnenschein Lab, Anatomy and cell biology, Tufts UniversityBoston, MA, USA; ^5^Laboratoire Ecologie and Evolution, École normale supérieure, Centre National de la Recherche ScientifiqueUMR 7625, Paris, France

**Keywords:** bottom-up, extended criticality, renormalization, levels of organization, singularity, organism

## Abstract

Biological thinking is structured by the notion of level of organization. We will show that this notion acquires a precise meaning in critical phenomena: they disrupt, by the appearance of infinite quantities, the mathematical (possibly equational) determination at a given level, when moving at an “higher” one. As a result, their analysis cannot be called genuinely bottom-up, even though it remains upward in a restricted sense. At the same time, criticality and related phenomena are very common in biology. Because of this, we claim that bottom-up approaches are not sufficient, in principle, to capture biological phenomena. In the second part of this paper, following (Bailly, 1991b), we discuss a strong criterium of level transition. The core idea of the criterium is to start from the breaking of the symmetries and determination at a “first” level in order to “move” at the others. If biological phenomena have multiple, *sustained* levels of organization in this sense, then they should be interpreted as *extended* critical transitions.

## 1 Introduction

From our point of view, the topic of this special issue, “Is life a globally critical phenomena, and if so why?” raises a question of *principles* in theoretical biology. Because of that, we think that this subject goes beyond the realm of biophysical models and concepts, which are mostly inherited from physics, and leads to questions that are proper to biological theoretizing.

There are at least two reasons for considering that biological matter can be considered as a globally critical phenomena. The first reason emerges from the question of the fundamental theoretical symmetries in biology. We examined this point in Longo and Montévil (2011), Longo et al., 2012; see also Bailly and Longo, 2011). We will quickly sum up some of our arguments right below for the sake of completeness, as they also constitute an answer to the question raised by this special topic. The second reason stems from the intuition that living systems have different “levels of organization.” This is the main aspect our paper deals with.

Let’s first summarize our proposal on the role that symmetries play in biology, by contrast with the situation in physics. In theoretical physics, symmetries are stable: they are the foundation on which the theories are grounded. It is fair to say that symmetries allow to constitute theoretical objects. Indeed, theoretical objects in physics are defined by their equational determination and the latter relies on the fact that, for various transformations (symmetries) applied to the equations and from the right, well-chosen points of view, the situation remains the *same* (it is invariant). This invariance is what we call the *genericity* of physical objects^1^. Besides, such an equational determination is valid also because it determines the *specific* trajectory followed by a given object, in principle a “geodetics.”

By contrast, we proposed in Longo and Montévil (2011), Longo et al. (2012), that in biological situations the relevant theoretical symmetries are not stable, but broken by the temporal flow. We proposed that this amounts to make the hypothesis that biological objects are specific: their theoretical symmetries change and they *become* defined/specified along (and by) their history. In other terms, the theoretical symmetries change with the flow of time. And, by considering that phase spaces are defined with respect to symmetries, we were lead to the conclusion that there is no stable phase space which would allow to capture or theoretically determine the trajectory of a biological object (Bailly and Longo, 2011; Longo et al., 2012).

Now, physical critical situations also appear, in particular, when the symmetries of a given object change^2^. However, critical situations still pop out from a pre-given phase space, where they appear as a singular point in a background of regular behaviors. By contrast, we claimed that in biology, criticality, in the above sense of symmetry changes, is pervasive, and not restricted to points in a predetermined phase space. We called such a situation with pervasive, non-punctual, symmetry changes, an *extended critical transition* (Bailly and Longo, 2011; Longo and Montévil, 2011; Longo et al., 2012). In short, see the references, critical transitions in biology are not confined to a point, as required by the mathematical treatment of physical criticality, but to a non-trivial interval of one or more control parameters. Such a situation, we argue, implies a major methodological change with respect to physical theoretizing.

As we mentioned earlier, there is a second reason to consider that organisms are in extended critical transitions. This reason is based on the notion of levels of organization and on the possibility (or not), to use bottom-up approaches in order to understand these systems. Theoretical accounts^3^ on living systems are diverse and these accounts crucially depend, in particular, on the scale and/or level of study the biologist focuses on (think, for instance, to a molecular approach compared to a tissular or an organismal one). However, when different accounts are meant to deal with what is supposed to be the same biological matter, we are faced with the question of pinpointing the relationships between these approaches, and the possibility to understand one scale/level of description in the terms of another, which could give the primacy to bottom-up approaches. The first part of this paper deals with this question. The second part deals with the question of the nature of the levels of organization in living systems and provides a strong definition of the change of level of organization. We will also explore some theoretical implications of the coexistence of several levels of organization in a living system.

First, it should be noticed that “scales” and “levels,” despite some laxity in the literature, are not commutable terms. Scales appear through *quantities* varying in magnitude; they can have the dimensionality of, e.g., space, time, energy, mass, etc. By contrast, levels of organization appear through *qualitative* changes between objects that are organized in a hierarchical manner (for examples atoms, molecules, organelles, cells, organs, organisms, etc.). As we will see below, these two notions of scales and levels of organization are not equivalent, especially in biology. As a first example of this difference, we can mention the allometric relationships. These relationships describe the change of certain quantities (such as the metabolic rate) through scales (usually measured by the mass), while the level of organization is kept fixed (usually, allometries are drawn at the level of organisms, see for example Savage et al., 2004).

In biology, scales are usually not seen as problematic, since they are mostly inherited from physically defined quantities. Contrariwise, the notion of levels of organization, while widely used, appears to be loosely defined, and its relevance in principle, beyond mere heuristics, is still a matter of debate (Bailly, 1991b; Brigandt and Love, 2008). Therefore, in the first part of the paper, we will remain neutral as to the notion of level of organization and will restrict ourselves to inter-scale theoretical relationships. To examine this point, we will make a detour through physics, as the conceptual stabilization of this field by mathematization makes it easier to clarify conceptual issues. We will then come back to the question of inter-level theoretical relationships, and to the question of their validity in principle.

## 2 Critical Phenomena and Bottom-Up Approaches

In this part we consider the question of the possibility and the modalities of understanding the whole as a combination of the parts. We will argue that critical phenomena are peculiar with this regard.

### 2.1 Bottom-up approaches in physics

Here, we do not intend to provide a comprehensive picture of bottom-up approaches in physics. On the contrary, we will focus on situations which are relevant for our purpose. In this field, the question is mainly that of the mathematical derivability of the upper-scale determination from the combination of interactions of the parts. The modalities of the integration of the parts will determine if and in what sense bottom-up approaches are used.

#### 2.1.1 A paradigmatic case: from molecules to thermodynamics

The main way to understand an upper-scale phenomenon via a lower-scale model is to use some form of statistical averages over the lower-scale properties, provided that the number of lower-scale entities is sufficient. The reconstruction of thermodynamics by statistical mechanics is one of the most (supposedly) paradigmatic examples of such a procedure^4^.

The principles of statistical mechanics are the following (see, for example, Sethna, 2006). We consider elementary objects (microscopic objects), which live in a given phase space and, therefore, have a state which is described in this phase space. A microstate is given by the state of all elementary objects. Each one of these elementary objects has a determined energy function, which depends on its state and on the states of other elementary objects (interactions). The sum of all these elementary energetic contributions defines the energy of the considered microstate. Energy, as a function of the state, is called a Hamiltonian (it is a function, not a value). The crucial hypothesis is that all microstates with the same energy have equal probability. Therefore, the macroscopic equilibrium state corresponds to the most numerous set of macroscopically similar microscopic states, at fixed energy (microcanonical ensemble). This leads to Boltzmann’s interpretation of entropy, where entropy is the logarithm of the number of state at fixed energy. The most probable state is, therefore, the one with the highest entropy. Then, the (inverse) temperature can be introduced as a quantity associated to energy (Lagrange multiplier), and the distribution of states follows by generic optimization principle (for a large number of particles). All statistical properties are then given by the partition function $Z=\sum_{s\in M}\exp\left(ℋ\left(s\right)/k_{b}T\right)$, where *M* are the microstates. The probability of each state is $\exp(ℋ(s)/k_{b}T)/Z$.

The point is that the distribution depends only on the Hamiltonian, and the conjugated variable, the temperature (when relevant, other parameters can be introduced). Moreover, the thermodynamical quantities can be obtained straightforwardly as averages, sums, variance, etc., from this distribution. Mathematically, this is elegantly obtained by elementary operations on *Z*: differentiation, application of the logarithm, multiplication by the inverse temperature, etc. For example, the macroscopic energy is the expected value of the energy of the microstates $\langle E\rangle =-\frac{\partial \ln Z}{\partial 1/k_{b}T},$ see any textbook or Sethna (2006) for other examples.

The probability of deviating from the most probable state decreases exponentially, depending on the number of lower-level entities (this result is known as the fluctuation theorem). Thus, here, the bottom-up approach corresponds to the determination of the macrostate by microscopic energies and is, in this case, mathematically achieved and controlled.

Notice that the elements “include,” in a sense, macroscopic or at least mesoscopics aspects in their mathematical description. The hallmark of this is the dependence on temperature. Similarly, when one approaches the microscopic level with classical mechanics, certain notions and hypotheses have to be introduced^5^. The main one are the thermodynamic limit (the assumption of an infinite number of particles leads to a coincidence of averages and macroscopic states) and the notion of ergodicity (that is a symmetry assumption between time average and phase space average). The latter allows to go from the properties of a trajectory to the properties of the phase space and vice versa. Therefore, in both cases, the elements are defined in such a way that they embed the “shadow” of macroscopic aspects. However, and this is the point we want to make, the mathematical situation here is so that it allows to fruitfully perform these audacious conceptual operations.

#### 2.1.2 When means fail: critical phase transitions

There are some cases in physics where approaches of the kind described above fail. This is particularly the case in some second-order phase transitions, in thermodynamics (Toulouse et al., 1977).

An example is as follows: a piece of iron can be considered as composed of a large number of elementary magnets at fixed positions. These elementary magnets tend to be in line with their neighbors (that is the lowest energy state), but temperature tends to break down this alignment (it increases the propensity to high energy states). Below a given temperature, the elementary magnets are predominantly aligned, and the piece of iron is globally magnetizable. Above this temperature, the thermic agitation is large enough to prevent the elementary magnets to be collectively aligned. The transition between these two behaviors (macroscopic order versus disorder) does not occur progressively but at a precise temperature, which defines the critical point. When approaching this point, the correlation lengths tend to infinity, which means that the elementary magnets fluctuate in an increasingly collective manner. At the critical point, there are fluctuations at every scale, which means that there is a tendency to obtain magnetic alignments of every size. Moreover, some physical quantities become infinite at the critical point (susceptibility to an external field, for example, which is a measure of the effect of an external field going from 0 to ± ∈).

But, can a system with fluctuation of any sizes be effectively described by averages?

Landau theory aims to do so. Landau’s strategy is the following: he assumes that we can obtain such a purely macroscopic account and then derives the consequences of this assumption. The latter leads to a first determination of the mean of considered variables, and allows to compute the local (in the sense of the correlation length) structure of the fluctuations near the mean^6^, when tending toward the critical point. If the means dominate the local behavior, then the approach based on them is valid. However, when the fluctuations dominate the local behavior, it implies that they dominate “local” behavior of arbitrary sizes, since we are tending toward the critical point, where the correlation length diverges. In the latter case the approach is therefore not consistent.

The distinction between the mean dominated and the fluctuation dominated situations is given by the Ginzburg criterion (a mathematical criterion, which depends, in particular, on the dimension of space; see Als-Nielsen and Birgeneau, 1977).

A related approach to phase transition is the mean-field theory. The basic idea of this approach is to consider microscopic interactions and to replace the non-linear (bilinear typically) interactions between particles by interactions with a global parameter representing their mean (e.g., ∑*S_i_S_j_* = ∑*S_i_S*). This usually leads to consistency equations (given by *S* = 〈*S_i_*〉). This approach, initiated by Landau and Ginzburg, is also clearly related to the validity of the macroscopic, mean parameter. Landau theory can also be technically assimilated to it (Landau theory is in this sense a mean-field theory)^7^.

Thus, for systems undergoing a second-order phase transition, we have a criterion which allows us to determine where bottom-up approaches (here, by the uses of macroscopic means) succeeds. In the preceding section, however, we said that the description by statistical mechanics converges nicely when the number of elementary objects increases. Why, then, is this description so badly broken here? The point is that the convergence toward the mean is based on a certain independence of the microscopic degrees of freedom, which essentially leads to a statistical convergence (central limit theorem). However, when the fluctuations dominate, we have a coherent mesoscopic structure (non-independence). When we approach the critical temperature and the thermodynamic limit simultaneously, we obtain a system whose statistical properties are dominated by fluctuations. Mathematically, this corresponds to singularities of the partition function at the critical point and thermodynamic limit, which, therefore, gives infinite quantities for certain statistical quantities (variance typically).

Systems undergoing second-order phase transition thus fall below the scope of classical bottom-up approaches, since our ability to understand the system (when its elementary components are put together) is undermined by divergences generated by their combination.

#### 2.1.3 Bottom-up vs. renormalization

We will now examine how systems at a critical point are understood. This will allow us to determine to what extent the theoretical approaches used for these systems can be considered as bottom-up, top-down, or something quite different.

In order to study critical phase transitions, renormalization methods^8^ are used. We can describe them as follows. One starts with a system described by a model at a given scale (which can be chosen arbitrarily; for instance, one can choose the resolution of the measurement apparatus). This model is composed of a set of parameters and of a function that determines the behavior of the system (e.g., the Hamiltonian). Instead of solving the model at this scale, as one usually does, one looks at the way the parameters and functions change when the system is described at an upper-scale. The way models change as a function of scales is formalized by a mathematical operator: the renormalization operator.

In usual cases (like para-ferromagnetic transitions), the model is asymptotically invariant by renormalization. This means that when considering larger scales, the models obtained converge toward a fixed point. Such an asymptotic invariance corresponds to scale invariance. In this case, the physical properties of the system are determined by the behavior of the operator in the neighborhood of the fixed point.

The conceptual meaning of renormalization in critical situations is the following. We cannot mathematically obtain what the combination of elementary constituents leads to, because it generates singularities (at the thermodynamic limit), or in other words, because this combination does not converge nicely. In terms of fluctuations, the situation is not tractable because the local behavior is dominated by fluctuations that occur also at scales above the scale considered (whatever the latter is). We can nevertheless consider a limited part of the system’s interactions, bounded by arbitrary scale cutoffs. This limited part of interactions is then integrated and the result constitutes one of the new elementary parts of a new model. We cannot know what these new elementary components exactly do (solve the model), but we can relate them to the rest of the determination of the system (transform the equations that determine the system). In short, since one cannot consider all interactions of the system’s elements, one consider a limited part of these interactions. Then, one mathematically simplify this part, in order to produce a new equational determination. This renormalized equations are, however, as complicated as the original one since we have an infinite number of degrees of freedom. In general, the operation is performed in order to *conserve the equational form of the determination*, but with renormalized parameters and variables.

If some of these parameters vanish asymptotically, when we iterate the procedure, then the fixed point is simpler than the original equation. This procedure can also be applied in the case where the situation is not critical. The result is a simplified determination which justifies the smooth behavior obtained by Landau theory, for example. On the contrary, in the case of critical phenomena, which are of interest to us here, the resulting determination (the fixed point) remains essentially as complex as the initial one: we do not lose the fluctuations in the process because, as we said earlier, they dominate the behavior of the system at all scales. Why, thus, is this operation still a tremendous progress toward the determination of the global behavior of the system under study? When we are at the fixed point, by definition, the iteration of renormalization does not change the equational determination. As we said earlier, iterating renormalization consists in taking more interactions into account. Therefore, when we consider the fixed point, we are considering a stabilized situation with respect to the contribution of supplementary interactions. When we add interactions to the fixed point, in its renormalization, the changes defined by the renormalization operator takes into account their effects (because we have a fixed point). Therefore, the renormalization operator is a description of these interactions. In this sense, asymptotically, *all* the interactions are images (copies) of the interactions that are taken into account in the renormalization of the fixed point. Therefore, we have an *explicit* account of *all the relevant* (large scales) interactions in the system^9^, even though this account is not and cannot be given by a description at a single scale, that is this account is not an *actual* combination of all the relevant interactions in the system.

Finally, it is worth emphasizing that one can consider different models as starting points (possibly but not exclusively at different scales), for if they have the same asymptotic behavior through renormalization, then they lead to the same physical properties and are grouped into a unique class of universality (there is an infinity of models in a class of universality since, among a plethora of others, the renormalized model at any scale can be indifferently considered as a starting point, Lesne, 2003).

One can consider renormalization methods as a bottom-up approach, given that the study of the system mainly depends on the starting point, which is the lower-scale model. However, this starting point is largely contingent and arbitrary, both because one can start from any scale as a minimal scale and because one can change the starting model as long as it remains in the same class of universality. It is in the generic properties of the universality class that the objectivity of renormalization lies.

The renormalization approach has a holistic flavor, in that the local situation at the critical point depends upon the global situation (the coherence structure). Such a coherence structure takes place because correlation lengths are infinite. More precisely the system is “so global” that we cannot combine its interactions entirely, we can only, but explicitly, find the form of the contributions of all (large scale) interactions.

The radically new aspect of renormalization methods is that one does not, *stricto sensu*, try to solve a model anymore, but rather to know the behavior of the transition from one model to an upper-scale model. In this respect, the system is studied at the level of a meta-model: what matters, *in fine*, is not the intra-model relationships, but the behavior through scales of this inter-model relationship. This meta-model allows to start from a subjective model (shaped by approximations and pragmatic constraints) and to reach, through asymptotic properties, objective knowledge about the class of universality and physical properties of the system.

Quantum field theories deal with situations which also require the use of renormalization methods (where they were actually first invented). The point is that, when we are considering more and more microscopic^10^ interactions we are faced with divergences (comparable to divergences of critical phenomena). This means that the behavior at a scale cannot be given, in this theory, by the contribution of objects of arbitrarily small space scale (this would disrupt the equational structure, by the appearance of infinities). However, we can handle a part of the interactions, and consider the stability and the transformations of the equational forms and “constants” when we look at more and more interactions. The possibility for theories to be renormalized is a condition of their theoretical validity, here. The point we want to emphasize is that the standard model handles three of the four fundamental physical forces in this manner, where there is no objective smallest, fundamental scale. On the contrary, the introduction of a peculiar small scale behavior is in opposition with the manner in which the theory understand microscopic phenomena (Zinn-Justin, 2007). Of course, this does not preclude paradigmatic changes, especially because the introduction of gravity leads to non-renormalizability (taking more interactions into account leads to a complexification of the equational form, by the introduction of new variables). In this sense, the current understanding of microscopic phenomena is *bottomless* in terms of small scale.

In short, renormalization allows to provide an explicit (and measurable) account of *all* relevant interactions in a system when the *actual combination* of all relevant interactions is mathematically impossible. In other words, from the point of view of the theoretical determination, the whole is not the sum of the parts (the sum diverges) but it can be understood by successive partial sums of its parts (which become symmetric to all partial sums of parts at large scales). By this and *in fine*, the whole is not understood by the *sum* of its parts but by *sums* of its parts. In the process, the modelization of the microscopic scale appears for a large part contingent and arbitrary (and renormalization allows to single out objective aspects from it, that is the invariants of the process). For these reasons, we consider renormalization methods at the edge of bottom-up strategies. These methods appear as a mean to go beyond standard bottom-up approaches while keeping a relatively bottom-up flavor (integration of interactions), though a considerably weakened one. In particular, renormalization in quantum field theory is associated with a *bottomless* situation (the small scale behavior cannot be integrated in the sense of an actual combination).

### 2.2 Consequences for biology

Now, what holds for physics does not necessarily hold for biology or, more precisely, does not need to describe “completely” the biological situation. Moreover, physical criticality implies that even a successful physicalism would not necessarily mean that we can understand the organism as an *actual* combination of the interactions between its parts (as usually claimed by adepts of bottom-up approaches).

#### 2.2.1 Physical criticality and the living state of matter

Like physical critical systems, biological systems present a complex structure of interactions involving different scales, both in space and time (see for example the case of the heart; Noble, 2002). Simple collective biological phenomena have experimentally been described as critical in – almost – the physical sense (see Mora and Bialek, 2011), for a review of some examples. If one considers the question of susceptibilities (sensibility to perturbations), biological transcriptome networks (see Shmulevich et al., 2005; Nykter et al., 2008), or hair cells (Camalet et al., 1999) provide good examples. This kind of structure of inter-scale correlations could explain why critical phenomena (more and more) seem pervasive in biology (see for example, Bailly and Longo, 2008, 2011; Werner, 2010).

Even molecular biology can provide a somewhat paradoxical example. Indeed, when we abandon the notion of program (and we have substantial reasons for that, see for example Longo and Tendero, 2007; Noble, 2008), and when we look naively at the experimental manipulations performed, we observe that a microscopic experimental manipulations (mutations by substitution, for example, concern structures measuring 3.3 Å) can lead to dramatic consequences concerning the whole organism (inasmuch it manages to survive). This implies a considerable amplification of such perturbations over spatial scales.

Thus, the difficulty found in critical physical situations may well be encountered also in the study of organisms. However, the latter is more difficult since the structure of coherence of an organism is heterogeneous and not generally scale-invariant (even though some of its aspects have approximate scale symmetry (see West, 2006 and the examples above). Here by heterogeneity, we mean that different parts of a system have to be described by different theoretical objects (e.g., cells, collagen matrix, capillaries, organs, etc.).

Another point is that the accounts of biological objects can be different at different scales. This seems to be a crucial difficulty in comparison with the theoretical leverage of scale invariance used for physical critical systems. A pragmatic way to overcome this scale dependence is to consider biological systems simultaneously at different scales. This seems indeed to be, *de facto*, the current approach: biology is a growing field of flourishing sub-disciplines (Brigandt and Love, 2008). This variety corresponds also to the use of different mathematical techniques (see Saetzler et al., 2011).

The difficulty, when we have critical phenomena in mind, is that the inter-scale relationships are fundamental while the pragmatic point of view tend to consider almost unrelated slices of the phenomena at different scales. From a more strictly biological point of view, following (Soto et al., 2008; Bailly and Longo, 2011), the circular coupling of lower and upper-scales is an essential feature of biological phenomena. This coupling is particularly relevant with respect to time, and when one considers its effect along the historical constitution of the organism, during development. Bizzarri et al. (2011) also insist that the mesoscopic aspects, in particular fractal-like structures are key to understand biological phenomena.

#### 2.2.2 Conclusion on the consequences for biology

Because of these very aspects of biological systems, the renormalization method cannot be used directly (except in some simple situations) and requires at least to be deeply transformed. We will further investigate the application of renormalization ideas to biology in a future article. Nevertheless, we can already notice that at least in some cases parts of biological systems behave like physical critical situations and more generally like singular, fractal-like structures. Thus, it is fair to assume that biological systems cannot be understood through ordinary bottom-up approaches.

### 2.3 Conclusion of section 2

In physics, we have shown that bottom-up (or upward) approaches can lead to at least two different situations. The first corresponds to a validity of the approach of the macroscopic system by usual statistical quantities. The second, however, corresponds to genuinely critical situations, where a system builds up a global structure of coherence. In this case, the direct composition of the interactions occurring in the system leads to divergences (when going to the thermodynamic limit). Therefore, the situation cannot be studied by the composition of all the interactions in a model. However, the renormalization method nevertheless allows to understand the *global* structure of the interactions because it handles a partial composition of interactions which is symmetric to other compositions of interactions, at larger scales (the sum of all this interactions remains intrinsically divergent). This is also combined with a certain contingency and arbitrariness of the initial model of the interactions (as long as it remains in the same class of universality).

The upward approach, for these reasons, can have a highly counter-intuitive nature in this situation. As we have observed, the determination of the system is not given by the *sum* of (the determination of) its parts but by *sums* of its parts (in an iterative way). Because certain biological situations have clear empirical critical signatures, the validity of bottom-up approaches in biology cannot be claimed, in full generality, to be stronger than this largely weakened version.

This, however, does not mean that biology can be understood by this form of upward understanding, and an even weaker form, if any, may be required. Indeed, biology seems to imply a finite class of heterogeneous, circularly coupled scales, which means, under the hypothesis of criticality, that several scales may be fundamentally relevant and that their co-determination may be a fundamental and constitutive aspect of biology.

## 3 Levels of Organizations

For the moment, we remained neutral as to whether there are levels of organization in biology, and if there are, what the nature of these levels could be. However, the question of bottom-up approaches in biology is typically framed in terms of *levels* of organization (Brigandt and Love, 2008), not only scales. The question of the nature of these theoretical levels is of prime importance to the development of a science of systems, as it seems to be the aim of the growing field of systems biology.

But, if, at first sight, the notion of level of organization seems intuitive, it appears that we still lack objective criteria allowing us to determine what should count as a level or not (Bailly, 1991b; Brigandt and Love, 2008). In particular, we would like to be able to distinguish between the complexification *within* a given level, and the *transition* between two levels. Indeed, at a given level, we can observe various degrees of complication, corresponding to an accumulation of objects (see below), or, mathematically, to an accumulation of degrees of freedoms or of iterations of a recursive function. This question is crucial theoretically, since, for example, there is no obvious reason why certain systems, argued to have fundamental biological relevance, should have an original structure of determination. For example, Piedrafita et al. (2010) argue that peculiar chemical systems, corresponding to a given circularity criterium, should be a (minimal) model of a fundamental aspect of biological organization. However, since the system is written and theoretically handled by usual reaction kinetic theory, it is not clear why the system should be biologically fundamental, and associated to a specific level of organization.

Here, we first aim at objectivizing the notion of level of organization, following mainly the work of Francis Bailly (Bailly et al., 1988;Bailly, 1991a,b; see also Bailly and Longo, 2011), adapted to our own understanding.

Let us first emphasize that, here, we understand a theoretical level of organization in a unusually strong sense. A level of organization will be, in the following, a fundamental level of theoretical determination of objects. Typically, in physics, this corresponds to a mathematical frame which determines the specific, theoretical trajectory of a given generic object. Note that, by contrast with Bitbol (2012), we do not approach the problem of levels of organization from (nor by a critique of) an ontological viewpoint. On the contrary, we are interested in the possibility, or not, to understand a physical or biological situation on the basis of a single level of description by mathematical means. When there is such a possibility, Occam’s Razor applies and we choose the relatively fundamental scale/level of description, from the proposed perspective, that is to say the one which enables to derive the others. From a mathematical point of view, the lower-scale/level of description is usually more detailed in the sense that the set of its elementary elements usually has more degrees of freedom than the set of elements described at a macroscopic level/scale. Moreover, if these components follow, say, classical trajectories, then the system follows a determined specific trajectory. The situation is, therefore, mathematically well determined, and other levels seem confined to have only an analytic relevance. We will see, however, that the situation is not that simple.

### 3.1 Complexification within a level

Following Bailly (1991b), complexification within a level of organization involves the combinatoric accumulation of objects defined and determined at this level. Accumulation of objects is made possible by the generativity of the determination. For example, classical electromagnetism accommodates any charge distribution and their movements. Indeed, most if not all theoretical structures of determination allow to accommodate combinations of arbitrarily large (finite) numbers of objects^11^. A fundamental example of such accumulation within a level is an increase in number of degrees of freedom, which leads to an accumulation of terms in a Hamiltonian, either independent or corresponding to interactions^12^.

Theoretical frames yield the pertinent observables and provide the relationships between finite quantities (number of objects, extensive or intensive quantities, time, etc.). This can be seen as the fundamental commensurability of the quantities which are involved. This commensurability precisely corresponds to the ability of a frame to provide the theoretical determination of the object described through these quantities. Now, it is crucial to keep in mind that the frame itself articulates these quantities, so that also “finiteness” should be understood relatively to it. For example, the integral of a function which is singular (infinite) at a given point can be finite, therefore if it is only this integral which is relevant and the systems determination is not disrupted.

When some *relevant* quantities become infinite, some fundamental operations can be degenerate (e.g., ∞ + ∞ = ∞) or undefined (e.g., ∞ − ∞ = ?). For example, one usually does not know, by the determination of the system we already have, what a physical system will do after a finite time blow up (a situation where the solution becomes infinite in finite time, and is, therefore, interpreted as a break down of the determination if there are physical reasons to consider that the considered quantity must remain finite). The crucial point is that, when no relevant infinite quantities are involved, the accumulation of terms does not change the structure of the theoretical frame. In quantitative terms, when no infinite quantity is involved, causes and effects remain commensurable (incommensurability would occur typically when the ratio between a cause and an effect becomes infinite).

### 3.2 Two types of infinity

It is worth emphasizing that at least two types of infinity should be distinguished. The first deals with the extensive properties (sizes or more precisely the number of objects) and occurs via the *accumulation* of objects within a level. The second deals with the intensive (so to speak “qualitative”) properties of objects and will appear to relate to a change of level. “Intensive” means that the given quantity, in a homogeneous system, is independent of the size of the system, and can, therefore, be considered to be a local property (we will come back to this point later). In particular, the ratio between two extensive properties is an intensive property. Typical extensive quantities are, in thermodynamics, the volume, the mass, the number of particles, …. On the contrary, intensive quantities are the temperature, concentrations, volumic masses, volumic, or massic thermic capacity, …

Infinite values have extremely different theoretical meanings whether associated to extensive or intensive quantities.

- With extensive infinity, the theoretical structure of the components is left unchanged, so the whole remains described by the same interactions between the parts. However, it is worth noticing that extensive infinity can transform a probabilistic determination of the system into a predictable determination, and thus can change the causal regime (typically, statistics may average). In parallel, extensive infinity can lead to the loss of time reversibility. These two modifications of the causal regime (determinism and time irreversibility) are typically encountered in the thermodynamical limit.
- By contrast, intensive infinity can disrupt the properties of the parts and, thus, changes the structure of determination of the system (such a disruption typically occurs, in thermodynamics of phase transitions, when the Ginzburg criterion is not met, typically when mean-field theory is not valid). Note that, in statistical physics, it is necessary to have extensive infinity for intensive infinity to be obtained (because of the analyticity of the finite size partition function).

It is also interesting to relate these two types of infinities to the question of the theoretical symmetries of a system. We should first keep in mind that symmetries have, in general, a “conservative” nature: they are transformations that can be inverted. However, physicists are not really interested in exact solutions. Even though they preserve all the symmetries of the initial hypotheses, they only allow to understand special cases and not general features. Let us consider an elementary example to illustrate this point. The classical relaxation equation $\frac{df}{dt}=-\frac{1}{\tau }f$ corresponds to a situation where the function and its derivative are proportional, which is a symmetry. Physically, this corresponds to a decay that is proportional to the magnitude of the quantity, so that all “small pieces” of the corresponding quantity decay independently (think of radioactive disintegration, for example). This symmetry is preserved during the trajectory and never allows the corresponding quantity to disappear completely since the function which verifies this symmetry is the exponential $\left(f\left(t\right)=f\left(0\right)\exp\left(-\frac{t}{\tau }\right)\right)$. However, after a time *t* ≫ τ, the corresponding quantity is extremely small and, for most practical purposes, it is negligible. By the use of infinite time limit, we can break the symmetry of this decreasing exponential and replace the value of *f* by its equilibrium value, 0. This allows to take into account the physically relevant behavior of the system for *t* ≫ τ, since the classical measure has a finite precision. This kind of reasoning is pervasive in physics. For example, renormalization is based on such considerations: it consists in separating relevant and irrelevant components, and the latter vanish at large scales, and, *in fine*, their objectivity is not guaranteed. A more sophisticated situation is the notion of symmetry breaking *sensu* (Strocchi, 2005), where infinity is required in order to decidedly, physically separate objects corresponding to the symmetry breaking (they cannot fluctuate from one macroscopic configuration to another). Another conceptually compelling example is the breaking of time reversibility of the Newtonian frame at the thermodynamic limit.

Last but not least, infinities should be handled carefully. In particular, when we consider two quantities that go to infinity, the way we approach this combination of infinities matters in general, and one cannot recklessly take any limit after the other (the limits do not commute without specific hypothesis). In this case, we usually have a discontinuity of the limit: the behavior at the limit is not the same and not even close to the behaviors near it (following another path toward the limit). For example, in the case of criticality, the thermodynamic limit (the number of objects *n* → ∞) and the singularity associated to criticality (*T* → *T_c_*) need to be taken jointly, via renormalization methods (Lesne, 2003). Qualitatively this is natural: the thermodynamic limit allows to define thermodynamic relationships, between macroscopic observables, but at the critical point fluctuations at all scale dominate the behavior of the system and a purely macroscopic description is not sufficient.

One should notice that infinite time, at least in a number of situations, can be assimilated to an extensive infinity (see, for example Lesne, 2003). Qualitatively, we have the same role of these two limits (they correspond to finding generic trajectories, either by a very long trajectory or through an accumulation of smaller trajectories). Statistically, making 1 random experiment on *n* independent similar objects, or making *n* successive experiments on a memoryless object are equivalent.

### 3.3 Transition between two levels

For a transition between two levels to occur (and not just a complexification within a level), it is necessary to have a change in the parts (or in their relationships). As we already mentioned, such a change seems to only occur through the apparition of intensive infinity; however, intensive infinity alone is not sufficient to break the determination of a given level.

Thermodynamics of second-order phase transition provides a good illustration of when intensive infinity can or cannot lead to a change of level. Landau theory handles critical systems with macroscopic (uniform) variables. This account constitutes a first level of determination, but it necessarily produces a singularity at the critical point. In particular, the fluctuations and correlation lengths will diverge. As a result, a first perturbative approach^13^ will discriminate situations where the mean dominates over the divergent fluctuations (in which case there is no change of level), and situations where it is the converse. When divergent fluctuations dominate, the macroscopic relationships blow up, and the theoretician must consider new, relevant objects (via the (semi-)group of renormalization). These objects allow to take into account the global structure of coherence associated to the domination of scale-free correlations. The theoretical determination, therefore, operates at a new level.

### 3.4 Criteria of transition

As a conclusion, we will consider that a transition from a level to another occurs when the two following conditions are met (Bailly, 1991b):

1. *Transition to infinity*: at least one intensive property that is relevant to the first level should be considered as tending to infinity (relevance here means that the given magnitude contributes to the determination of the objects).
2. *Change in relevant objects*: the fact that the magnitude tends to infinity should make obsolete the empirical and theoretical determinations of the objects. This should introduce new, relevant objects in the system’s determination; these objects will be associated to the new level.

We want to emphasize that this criterion has two fundamental strengths. First, it leads to *observable* consequences: the divergence of intensive properties. Second, it is based on a break down of the theoretical determination of the first level and not on its invalidity for extrinsic reasons. Both of these aspects allow to objectivize the understanding of a situation as constituted by multiple levels of organization.

## 4 Application to Biology

On the basis of the former analysis, we can now discuss levels of organization for biological systems, and the theoretical consequences of the way our definition leads to consider them.

### 4.1 Biological levels of organization as a hypothesis

Biological functions typically tie the parts together in an integrated whole. This justifies that biological functions can be intuitively associated with changes of levels of organization.

Therefore, let’s first start with the idea that there are several levels of organization within an organism (we will discuss this intuition below) and take this as an assumption. Because an organism, in general, experiences a range of internal and external conditions rather than being confined to a single point, the multiplicity of levels associated to the considered organism should be obtained within an interval of viability parameters (or a dense subset of it). Following the aforementioned criteria, the changes of levels of organization are associated to singularities. Therefore, criticality (understood here as singularities in the determination) should be obtained within a dense range of parameters, rather than at a single critical point as in usual physical situations. The intuition of multiple levels thus drives us to the hypothesis of an extended criticality of organisms (see also Bailly, 1991a; Bailly and Longo, 2008). In particular, a small effect at a given level can have incommensurable consequences and, therefore, access to an upper level (criterion of intensive infinity) within a dense range of a given parameter. This is in particular exemplified by susceptibilities which become infinite and describe the response to an external perturbation.

Reciprocally, it is clear that if organisms show *extended* criticality (that is, in the sense of symmetry changes within a dense range of parameters, *and* with a condition on the strength of the associated fluctuations), the multiplicity of levels of organization obtained in the extended critical transition is robust. Integration and regulation, within and by an organism, allow the global robustness in spite of the cascades of instabilities and susceptibilities proper to the continual critical transitions of extended criticality.

### 4.2 Fractals and functions

Let’s consider the hypothesis that biological levels of organization in the above sense are associated to biological functions.

Bailly et al. (1988) defended the thesis that the integration of parts within a whole is achieved, in particular, through fractal structures, because fractal structures play a fundamental role in exchanges between different media. In our current framework, fractal structures seems indeed to be the simplest structure associated to singularity. It is this singular situation which fulfills our criteria of level transition.

Reviewing several biological cases, they made the following hypothesis:

1. to any vital function defined at a given level within an organism (macromolecule, cell, organism), corresponds one structure (active sites, organelles, organs) exhibiting at least one fractal dimension associated to this function.
2. reciprocally, to any fractal dimension of a structure, corresponds a vital function, which integrates this structure in a whole.
3. moreover, the correspondence between the fractal dimension and the functionality is to be linked with the transition between levels of organization, as fractality enables to make compatible properties that have to be both singular and homogeneous (see below).

Bailly et al. (1988) then proposed to explain the occurrence of fractal structures in organisms by the satisfying of three constraints:

*Tendency to maximal proliferation*: for instance, the maximization of an exchange surface (to enhance the efficiency and precision of exchanges). This tendency of unbounded growth can be understood by a transition to the infinite limit.
*Steric constraint*: the living system is bounded (because of other developmental or evolutionary constraints).
*Homogeneity*: solution structures should be homogeneous, by which we mean that the singularities should not describe the neighborhood of a point but have to be spread in the relevant space. This constraint excludes solutions which fulfill only the two preceding criteria, such as limit points or surface points, where the asymptotic limits would play a particular role. By contrast, fractal solutions are homogeneous (in their singular behavior).

Examples can be found, for instance, in Bassingthwaighte et al. (1994), West (2006). Typically surfaces of organs or organelles tend to have fractal shapes, with infinite surfaces at the mathematical limit. It allows them to have a very high exchange surface with a limited volume. Now, singular behavior are also observed in temporal structures, for example heart rhythm. The latter correspond to the formation of a proper temporal structure of coherence, with a unity of the organism extended overtime.

In the context of an organism, these structures are associated to the functionality of the organs, and participate in constituting the organism as an upper level of organization. The organism is therefore not understood as a macroscopic combination of parts, but as constituted by the combination of multiscale, singular structures (which can interact at various scales).

### 4.3 Non-genericity of parts

The conceptual frame we describe leads here to a crucial problem in understanding biological systems. If they are organized in levels, then, in some cases, perturbations can propagate through scales and levels. Thus, if living systems are in extended critical transitions, then it is expected that all levels are always “coupled.” This coupling, on one hand, stabilizes (i.e., it contributes to setting the margin of viability or of the extend interval of criticality of other levels), on the other it may destabilize the other levels. For example, even objects at the lowest levels (say, macromolecules) do not necessarily follow specific trajectories *in vivo*, that is to say “effective” trajectories (in the system), described by the symmetries at *that level*. As a consequence, parts do not necessarily exhibit stable enough effective (i.e., in the system) symmetries for us to observe, since these symmetries may be broken at the points of level transition and by level interactions. Note also that certain parts are themselves in extended critical transitions (cells, typically).

A simple example of this feedback of extended criticality on the effective trajectories of parts (which are stable *in vitro*) is the trajectory of the structure of *DNA* of living organisms along evolution. The evolutionary trajectory of this macromolecule is, in particular, determined by the ability of the organism as such to survive and reproduce in its environment (Longo et al., 2012).

However, let’s assume that in some cases we can first consider a contingent determination at a level to obtain generic parts. The determination is contingent in that it would be local in time and could be limited, for example, to some cellular types, or some cellular history. In particular, this can be the case if we suppose that the rhythm of symmetry breaking is slow enough. Then we would obtain approximately a situation close to usual criticality with level transitions.

Now, with non-generic parts, it is through the stabilization associated to the organism structure that a relative stability of the structure of determination (that is to say, a relative genericity) of parts can be obtained. For instance, parts are maintained in their viability zones (by preventing ischemia, providing nutrients, etc.), their proliferation is tempered (Sonnenschein and Soto, 1999), etc. Also, in general, the determination of cells, for example as given by huge changes like differentiation, is provided in a coupling with the organism (Soto et al., 2008).

## Conclusion

We have seen that bottom-up approaches, understood as the way the parts combine to form the determination of the whole can have at least two decidedly different forms in physics. The first, which is a genuine bottom-up method, corresponds to the situation where the determination of the whole can be obtained directly by the combination of the contributions of its parts. The second, where bottom-up approaches reach their limit, corresponds to a situation where the determination of the whole diverges and where the formation of global coherent structures takes place. In this case, the theoretical and mathematical way to understand the phenomenon consists in considering partial combinations of contributions of parts to the change of the equational determination. In physical critical phenomena, the global structure is thus resolved because all the large scale contributions are *symmetrical*.

As for biology, it seems to us that the notion of level of organization is well grounded if a transition between levels corresponds to, at least, a non-genuine bottom-up approach. Following Bailly (1991b), we gave a criterion allowing to determine when one can consider that there is a breaking of the determination at one level, which, therefore, corresponds to a transition between two levels. This criterion is inspired by the theory of critical phenomena, and is based on the idea of intensive property tending toward infinity breaks the structure of determination. In biology, such singularities are achieved, among other, by fractal structures (for instance, the liver tends to have an infinite volumic surface of endoplasmic reticulum). Fractal structures appear to be connective devices (for instance membranes) that link parts together in an integrated whole. Singular behaviors are also encountered in temporal and spatio-temporal structures, which can be approximately described by physical methods. We expect, however, that the genuine theoretical scale symmetry of these phenomena are not as stable as in physics of critical phenomena (where the invariance of the exponent is sharp).

We have seen that assuming that living systems are organized in different levels, in the sense of Bailly (1991b), naturally leads to consider that living systems are in extended critical situation. Criticality here means that we have strong singularities so that several levels can coexist (for example, when the Ginzburg criterion shows that the mean-field approach fails).

Now, as we said in introduction, extended criticality has at least two features: continual symmetry changes and different levels of organization. Even though this two features often go together as far as physical criticality is concerned, they do not, emph stricto sensu, imply each other. For instance, in the case where Ginzburg criterion legitimates mean-field approaches (*d* > 4), we have a symmetry change without a strong breaking of the structure of the determination. In our view, these two aspects of extended criticality are complementary and jointly contribute to an understanding of biological organisms.

Extended criticality proposes to describe theoretical objects that have very unusual features in comparison with physical objects. The changes of their theoretical symmetries mean that they are constituted by their history (which is not, *stricto sensu*, a trajectory because the phase space is defined in this process). At the same time, these changes are associated to and allowed by critical singularities, which lead to the continuous formation of several levels of organization. The latter are not understood as embedded in each other, like Russian dolls, but, on the contrary, levels in the sense proposed here should be understood as an inter-scale structure, following the coupling of the very nature of the parts that concur at the structure of coherence of organisms.

## Conflict of Interest Statement

The authors declare that the research was conducted in the absence of any commercial or financial relationships that could be construed as a potential conflict of interest.

## Footnotes

^1^The fall of Newton’s apple is a pictural representation of that: an apple and a planet are the same object (i.e., they are generic) as regards both mechanics and gravitation, but with different parameter’s value (different masses in this case).

^2^Indeed, the theoretical determination relies on the available, relevant symmetries, and their change means either that a new quantity appears (via fluctuations), or on the contrary that a relevant quantity vanishes and relaxes its constraints on the system (which again leads to fluctuations).

^3^We purposely avoid the term “theory” here as theoretical accounts in biology do not (yet?) yield fully integrated theories (Brigandt and Love, 2008).

^4^Note that this example has been challenged, notably as soon as 1961 by Feyerabend (1985), see Cat (2007) for a review.

^5^Because of the novelty of concepts involved in the analytic process, it is tempting to view this process as a unification rather than a reduction. In any cases, see Cat (2007) for a review of the philosophical positions on unification.

^6^It means that we are considering the fluctuations from this mean. The approximation signifies that we are not considering how fluctuations can interact with each other and build up even stronger fluctuations.

^7^Paradoxically, it is precisely in order to study criticality that mean-field approaches where developed. They thus playa role in the understanding of phase transitions, as a first, technically unsophisticated approach, and, of course, are even more useful where they are valid – in our example, in dimensions four and above (see Als-Nielsen and Birgeneau, 1977).

^8^These methods were first used in quantum physics and were successfully transposed in the field of criticality by Wilson, who was awarded the 1982 Nobel Prize in Physics for this feat. This subject is presented, for example, in Toulouse et al. (1977).

^9^Of course in practice, approximations are usually performed.

^10^The nature of divergences in this domain can be diverse, but we are interested in going from small scales to large scales here. Notice that small scale, here, is equivalent to large energy. The reason for this is that the quantum momentum is the spatial derivation operator (associated with the constant *h*). A small scale behavior means a behavior with a high derivative (e.g., $\frac{d\sin\left(x∕\lambda \right)}{dx}=\cos\left(x∕\lambda \right)∕\lambda$) so that the energy is high. From the field point of view, the classical potential diverge when we are going toward the punctual source of the field.

^11^At least in principle, the analytical tractability in practice depend in particular on the apparition of simplifying symmetries.

^12^It is noteworthy that such combinations are usually associated to a linear aspect (the addition of terms in the Hamiltonian, the superposition of states in quantum mechanics and the corresponding unitarity of the Hamiltonian operator, …) and of a non-linearity, corresponding to interactions in the Hamiltonian or measurement in quantum mechanics.

^13^That is to say, an approach where we start from the Landau theory but try to specify it by considering fluctuations which do not change the mean behavior of the system, obtained in landau theory.
